# Surface Topography and Tolerance Quality Evaluation of Polymer Gears Using Non-Contact 3D Scanning Method

**DOI:** 10.3390/ma19071324

**Published:** 2026-03-26

**Authors:** Enis Muratović, Adis J. Muminović, Łukasz Gierz, Ilyas Smailov, Maciej Sydor, Edin Dizdarević, Nedim Pervan, Muamer Delić

**Affiliations:** 1 Department of Mechanical Design, Faculty of Mechanical Engineering, University of Sarajevo, 71000 Sarajevo, Bosnia and Herzegovina; muratovic@mef.unsa.ba (E.M.); pervan@mef.unsa.ba (N.P.);; 2 Institute of Machine Design, Faculty of Mechanical Engineering, Poznań University of Technology, 60-965 Poznań, Poland; 3 Institute of Energy and Mechanical Engineering Named After A. Burkitbayev, Kazakh National Research Technical University, Almaty 050013, Kazakhstan; ilyas.smailov.99@mail.ru; 4 Department of Woodworking and Fundamentals of Machine Design, Faculty of Forestry and Wood Technology, Poznań University of Life Sciences, 60-637 Poznań, Poland; 5 Institute of Safety and Quality Engineering, Faculty of Engineering Management, Poznań University of Technology, 60-965 Poznań, Poland; 6 CNT–Center for Advanced Technologies in Sarajevo, 71000 Sarajevo, Bosnia and Herzegovina; edin.d@cnt.ba

**Keywords:** involute gears, polymer gears, surface topography, tolerance grade, hobbing, additive manufacturing, structured-light metrology, quality control, adaptive tooling, automotive assembly

## Abstract

The shift toward lightweight powertrain architectures necessitates a detailed characterization of polymer gears to verify their efficiency and durability. This study investigated the effectiveness of non-contact structured-light 3D scanning for evaluating the surface topography and dimensional tolerance quality of polymer gears produced via distinct manufacturing technologies. A structured-light 3D scanner was used to capture dense point clouds (exceeding 6 million points) of gears produced by three methods: conventional hobbing (POM-C), Material Extrusion (MEX) with carbon fiber reinforcement, and Selective Laser Sintering (SLS). The manufactured parts were compared against the nominal Computer Aided Design (CAD) models to evaluate their geometrical deviations in accordance with DIN 3961 and surface roughness parameters per ISO 25178. The experimental results revealed a consistent ranking of manufacturing quality. The conventionally hobbed POM-C gear exhibited superior precision, achieving DIN quality grades of Q9–Q10 and the smoothest surface finish (S_a_ = 5.0 µm). Among additive manufacturing techniques, SLS-printed PA 12 showed intermediate quality (Q11, *S*_a_ = 12 µm), whereas MEX-printed PPS-CF exhibited significant deviations (exceeding Q12) and the highest surface irregularity (S_a_ = 25 µm) due to stair-stepping effects. These findings indicate that while additive manufacturing offers geometric flexibility, conventional hobbing retains a decisive advantage in dimensional precision. The optical scanning methodology demonstrated here constitutes an efficient metrological framework for gear quality control, with potential applications extending to the quality assurance of additively manufactured adaptive fixtures and assembly tooling, including automotive assembly operations.

## 1. Introduction

Advanced polymers are increasingly integrated into modern transmission systems due to their superior strength-to-weight ratios and versatile manufacturability, enabling the production of complex geometries with reduced inertial mass [1]. The distinct advantages of polymer gears over traditional metallic counterparts have established them as a critical area of study [2,3]. In addition to mass reduction, polymer gears offer favorable noise and vibration characteristics, dry-running capability, corrosion resistance, and chemical inertness, enabling implementation across a wide range of operational settings [4]. While representing a state-of-the-art technology, their inherently lower mechanical strength and limited thermal stability currently restrict their use to moderate load applications [5]. Furthermore, polymer gears exhibit a broad spectrum of failure modes, which have a decisive impact on material selection and are among the most pivotal considerations in the design process, thereby requiring engineering experience and extensive experimental testing programs [6]. Consequently, the limited availability of requisite material data, coupled with the challenges of adapting traditional gear design methodologies to polymers, remains a central focus of ongoing studies [7].

As the demand for polymer gears grows, several manufacturing methods have become essential for meeting production-scale quality requirements. Injection molding is the primary choice for high-volume series due to its per-unit cost-effectiveness and the significant design flexibility it affords [8]. However, this process necessitates precise control of molding parameters to mitigate shrinkage and distortion during the material-cooling phase, both of which critically influence final gear quality. Conversely, conventional machining—specifically hobbing—is frequently employed for smaller production quantities [9]. Despite being constrained by cutting tool geometries, the hobbing process remains an efficient solution for larger modules, particularly when achieving tighter tolerances is a priority [10].

However, precise gear cutting necessitates significant material removal, thereby increasing overall polymer waste. Recent advancements in additive manufacturing (AM) have enabled the fabrication of polymer gears through various 3D printing techniques [11,12,13]. While often characterized by longer production cycles, AM facilitates advanced gear designs—including complex micro-textures and optimized material utilization—that overcome the inherent limitations of subtractive or formative methods [14]. Yet, despite the growing adoption of 3D printing for lightweight gear design, its load-bearing capacity and dimensional accuracy have yet to consistently meet the requirements of industrial applications.

AM is frequently utilized in the development of adaptive fixtures and modular locating systems, which have become critical for efficient vehicle body and powertrain assembly. Unlike conventional steel jigs—typically machined for a single configuration—additively manufactured fixtures can be geometrically tailored to individual part variants without retooling, offering a decisive advantage in mixed-model automotive assembly [15,16]. However, the structural and dimensional limitations of polymer-based additive processes, such as layer anisotropy, residual thermal stress, and surface staircase effects, impose constraints on achievable form accuracy that must be quantified before deployment in safety-relevant assembly operations [17].

In this context, the non-contact optical metrology approach presented in this study provides a methodological framework for incoming inspection of such tooling elements. Surface topography parameters characterizing the contact interfaces and tolerance grades reflecting the overall form accuracy can be rapidly extracted from a single scanning session, eliminating the need for component-specific tactile probing strategies [18,19]. Consequently, these findings have direct implications for the quality assurance of additively manufactured adaptive tooling within automotive assembly environments.

Beyond the geometrical and material selection, the functional design of polymer gears encompasses the interface topography, which serves as a critical predictor of efficiency and service life [20]. From this perspective, the choice of material and production method is of paramount importance; all engineered surfaces exhibit roughness at some scale, regardless of the fabrication process [21]. The interplay between surface topography—comprising roughness, microtexture, and waviness—and polymer mechanical properties dictates the reliability of gear systems [22]. Effective management of these variables mitigates localized stress, wear, and premature fatigue, thereby ensuring operational integrity. Consequently, surface roughness is a core design characteristic intrinsically linked to dynamic properties and is essential for accurate lifespan prediction, fault diagnosis, and risk mitigation. A standard set of 3D surface roughness parameters, compliant with ISO 25178, is typically evaluated using contact, non-contact, or comparison routines, facilitating the optimization of surface functional performance [23]. With the advancement of structured-light methods toward high-fidelity reconstruction of macro- and microstructures, general-purpose 3D scanners are evolving into proficient tools for surface analysis [24]. However, current technology necessitates the implementation of specialized digital filters to extract surface roughness by isolating it from underlying waviness and form curvature [25].

At present, accepted practices in gear inspection rely predominantly on tactile methods, which limit the assessment of tolerance grade and geometric deviations to predefined two-dimensional (2D) profiles and discrete measurement traces. Consequently, this approach neglects the functional characteristics of the entire gear flank [26]. However, alignment with the objectives of precision engineering necessitates a more rigorous dimensional inspection based on areal data. This enables a data-driven evaluation metric, particularly through the use of a point cloud captured via optical methods [27]. Optical measurements offer significant speed advantages over traditional contact methods and facilitate the characterization of fine-pitch gears that are otherwise difficult to access with physical probes. Furthermore, the areal measurements provide comprehensive evaluation of geometric parameters and their associated ranges for defining quality grades. According to DIN 3961, tolerance grades are classified into 13 categories, from Q0 (highest accuracy) to Q12 (lowest) [28]. Quantitative data on attainable tolerances across various manufacturing methods can identify necessary refinements in design and fabrication control, thereby enhancing optimization strategies and improving the overall mechanical and thermal performance of polymer gear drives [29].

Non-contact 3D optical scanning has become a key tool in gear metrology, enabling rapid and detailed acquisition of surface geometry data. These techniques generate high-density point clouds at rates exceeding millions of points per second, significantly outperforming conventional contact-based methods in terms of inspection throughput [30]. They are particularly advantageous for gears with complex geometries—such as polymer gears—where the precise representation of both macro-geometry and surface topography is essential [31]. The typical metrological workflow encompasses point cloud acquisition, surface reconstruction, and subsequent comparison with the nominal CAD model to geometric deviations and tolerances. Furthermore, detailed surface topography measurements facilitate the analysis of real contact areas, wear mechanisms, and the influence of specific manufacturing processes on gear durability. Vision-based non-contact techniques additionally enable high-precision assessment of wear and failure modes while circumventing the systematic errors inherent to tactile measurements [32].

Furthermore, integrating non-contact metrology with digital tools—such as augmented reality—enhances both inspection and design-optimization workflows. The acquired data are applicable to tolerance classification, reverse engineering, vibro-acoustic root cause analysis, and the refinement of gear tooth contact models beyond standard tolerance classification [33]. Despite their high efficiency, non-contact methods suffer from reduced measurement accuracy. To enhance precision, advanced computational procedures are implemented, including point cloud alignment with CAD models, reconstruction of actual tooth surfaces, and NURBS-based modeling. While point cloud filtering improves the estimation of contact patterns and transmission errors, it is important to note that such filtering protocols influence the resulting gear accuracy classification [34].

This study aims to develop and validate a structured-light, non-contact 3D scanning method for the optical measurement of polymer gears. Particular emphasis is placed on assessing the influence of point cloud density and data relevance on the evaluation of surface roughness parameters and dimensional tolerance quality. To demonstrate the method’s applicability across diverse manufacturing strategies, the study analyzes and compares the surface topography configurations and geometric deviations of gears produced via three distinct technologies, conventionally hobbed polyoxymethylene copolymer (POM-C), MEX-printed carbon fiber reinforced polyphenylene sulfide (PPS-CF), and SLS-printed polyamide 12 (PA 12), thereby additionally highlighting variations in manufacturing quality. To the author’s knowledge, this work represents one of the first attempts to utilize a 3D scanning framework for the simultaneous evaluation of surface roughness and tolerance grades in polymer gears. Consequently, this research enables a novel optical methodology for the integrated assessment of topography and tolerance ratings derived from a single point cloud, while providing critical insights into the manufacturing fidelity of additive versus subtractive technologies.

## 2. Materials and Methods

### 2.1. Specimen Manufacturing

The polymer gear specimens examined in this study were fabricated using representative materials and production technologies, specifically conventional hobbing and additive manufacturing. While multiple gears were produced, a single representative specimen from each manufacturing batch was selected for comprehensive metrological characterization. To ensure statistical significance, 30 measurements were performed on each of the 17 gear teeth, resulting in a dataset of 510 measurements per gear. This procedure was repeated five times, with the arithmetic mean utilized for the final tooth evaluation—a frequency considered sufficient for tolerance quality assessment. Although comprehensive process repeatability studies typically require larger sample sizes, the current work focuses on demonstrating the feasibility of the proposed 3D scanning framework for surface roughness and tolerance grade evaluation. Given the consistent use of standardized polymer stocks and optimized production parameters, the data stability observed during this preliminary phase is considered sufficient for methodological validation.

To accurately represent the ongoing progress in additive manufacturing (AM)—specifically within the context of gear design—two distinct methods were employed: Material Extrusion (MEX) and Selective Laser Sintering (SLS). Conventional machining was performed on extruded polyoxymethylene copolymer (POM-C) stock rods using a profile A hob cutter, designed for involute gears with a 20° pressure angle [35]. While conventional machining of steel gears per ISO 53 [35] typically yields high dimensional accuracy, polymer gears are expected to exhibit greater deviations due to their lower thermal stability [36]. The MEX process was utilized to fabricate carbon fiber-reinforced polyphenylene sulfide (PPS-CF) gears using an Ultimaker Factor 4 system (UltiMaker B.V., Utrecht, The Netherlands). Additionally, an SLS system (Formlabs Fuse 1, Formlabs Inc., Somerville, MA, USA) was used to produce polyamide 12 (PA 12) gear specimens. The primary fabrication routes for all polymer gear specimens are compared in Table 1.

The primary physical and geometric properties, alongside their associated test procedures, are detailed in Table 2 [37,38,39]. As tabulated, flexural and tensile-related properties of PPS-CF material are specified following the print orientation and are structured in the data for the *xy* (flat) plane, *yz* (side) plane, and *z* (upright) direction.

The data presented in Table 2, associated with three distinct production methods, are expected to exhibit diverse relationships regarding mechanical and thermal properties and dimensional and surface deviations. For instance, the hobbing process may result in reduced dimensional accuracy and tooth thickness deviations due to tool-induced deflections, which are directly correlated to the tensile modulus of the polymer [55]. Similarly, the low thermal resistance of POM-C often leads to surface deviations caused by localized melting and softening [56]. Furthermore, the appearance of machining marks—closely linked to material hardness—is anticipated.

Regarding the MEX method, the cooling of the polymer post-extrusion induces dimensional shrinkage and warping, while the viscoelastic behavior of the molten polymer can lead to layer distortions and subsequent inaccuracies [57,58]. Additionally, as interlayer adhesion forces may limit mechanical bonding, the final surface often exhibits prominent layer lines that significantly impact surface roughness. The elastic properties of the PPS-CF material may also cause deformation of the base layers during the printing process. Similarly, the partial melting and fusion of PA 12 powder during the SLS process often result in a porous, rough surface texture, particularly at high melt viscosities. Finally, the cooling of sintered PA 12 causes shrinkage and warping, leading to geometric distortions, while its relatively low stiffness may result in part deformation while in a high-temperature state.

Manufacturing parameters for MEX technology were configured based on the manufacturer’s recommendations using the ‘Engineering’ profile. Key settings included a 100% infill density, a layer height of 0.2001 mm, a wall thickness of 2.2 mm, a print speed of 80 mm/s, and a printing temperature of 320 °C. While numerous additional variables can be adjusted within the UltiMaker Cura slicing 5.12 software, these parameters are automatically optimized when selecting the PPS-CF material, the Print Core HT 0.6, and the ‘Engineering’ profile. Regarding the SLS technology, manufacturing parameters are inherently defined by the specific hardware and material system. Consequently, default settings for the Formlabs Fuse 1 were applied during fabrication, including a layer thickness of 110 µm and a 200 µm Ytterbium fiber laser (FWHM). All auxiliary parameters were managed via the default (v5.3) printing profile. For both AM technologies, the build orientation was defined such that the gear shaft axis remained perpendicular to the build plate.

The manufacturing parameters and material systems examined in this study are representative of those employed in the production of functional polymer components for industrial assembly tooling. MEX with carbon fiber-reinforced polymer feedstock and SLS with polyamide 12 powder are among the most widely adopted processes for rapid fabrication of adaptive fixtures, locating nests, and modular assembly supports in automotive manufacturing environments. Consequently, characterizing their surface topography and dimensional accuracy within a unified metrological framework is of critical importance—not solely in the context of gear applications, but more broadly for any polymer-based structural element in which contact surface quality and form accuracy are functionally significant.

### 2.2. Preprocessing and Measurement

**Figure 1:**
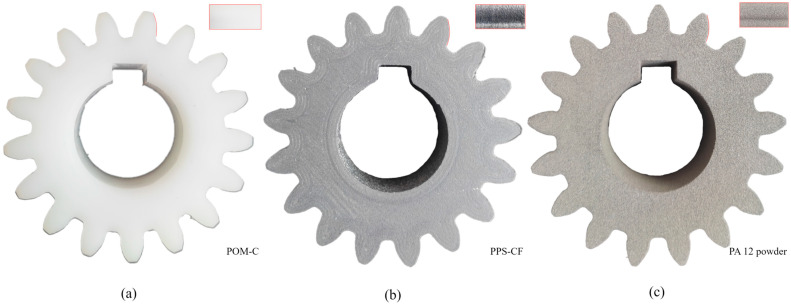
Representative polymer gear specimens produced via different manufacturing technologies: (**a**) conventionally hobbed POM-C; (**b**) MEX-printed PPS-CF; and (**c**) SLS-printed PA 12.

An optical method using structured light, fringe projection, and blue illumination is employed to measure the gear samples. A high-precision 3D scanner (GOM Scan 1, Carl Zeiss GOM Metrology GmbH, Braunschweig, Germany) was used to obtain areal measurements of polymer gears, producing a point cloud with over 6 million points per scan, enabling high resolution of even the finest geometrical details. Figure 1 shows the measured specimens. To illustrate the diffractive patterns of diverse manufacturing methods, the distinct topographies of tooth flank surfaces are shown in Figure 1.

Technical specifications, measurement parameters, and metrological performance data for the employed 3D scanner are detailed in Table 3.

**Figure 2:**
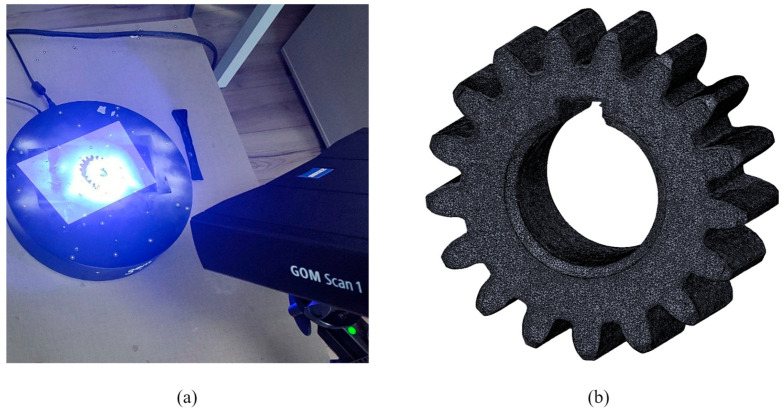
Areal measurements of polymer gears: (**a**) scanning setup; (**b**) 3D mesh.

The experimental setup for 3D scanning is illustrated in Figure 2a. The measurement procedure involves positioning the gear specimens on a motorized turntable to facilitate multi-view data acquisition [59]. These individual scan segments are subsequently registered and merged to generate a complete surface mesh, which is then exported in the Standard Tessellation Language (STL) format, as depicted in Figure 2b.

STL-formatted measurement data from the polymer gears are utilized both for surface roughness evaluation and the quantification of geometric deviations via point cloud analysis. Since gear performance is governed by tooth interaction, characterizing the flank topography requires isolating the relevant areal data into a single-flank geometry. This extraction is achieved through a localized coordinate transformation and a customized computational pipeline designed to isolate the topological features of individual tooth flanks [60]. Leveraging the analytical mechanics of gear involutometry, the coordinates of the tooth flank surface are then defined in a half-thickness representation relative to the tooth centerline.

As a prerequisite for coordinate transformation, the user-defined framework requires raw *x*, *y*, and *z* data in ASCII format, exported directly from a 3D mesh. Consequently, a single tooth flank geometry is represented by 126,622 surface points. These points are further processed to determine surface roughness parameters, by implementing a secondary local coordinate transformation to each point—effectively filtering out the involute curvature of the tooth flank. For comprehensive details regarding computational contact analysis, readers are referred to the authors’ prior work [61]. Utilizing raw areal coordinates in ASCII format facilitates subsequent surface topography and tolerance quality evaluation. Furthermore, the use of raw data ensures compatibility across different computational frameworks; its universal nature allows for seamless integration with diverse software interfaces, making the methodology accessible to various scientific and engineering disciplines. The initial coordinate transformation from a gear geometry at a defined radius to a half-width of a single tooth flank is described in Equation (1) [62]:

(1) $$T(x,y,z)=2\cdot r(x,y,z)\left[\frac{T_{1}\left(x,y,z\right)}{2\cdot R_{1}(x,y,z)}+{inv\phi }_{1}\left[R_{1}(x,y,z)\right]-inv\phi \left[r(x,y,z)\right]\right]$$

where T(x,y,z) represents the tooth semi-width at a specific coordinate, r(x,y,z) is the local radius relative to the gear center, and $T_{1}(x,y,z)$ denotes the reference tooth thickness at a given point. $R_{1}(x,y,z)$ is the radius of curvature at the corresponding coordinate, while $\phi_{1}(R_{1})$ represent the pressure angles at radii $R_{1}(x,y,z)$, and ϕ(r), respectively. The involute function, inv, is fundamental to the second coordinate transformation. This transformation is expressed via the vectorial angle θ, which is determined by the relative position of the coordinates at the base circle and the projection of the involute’s vectorial angle for any arbitrary point, as defined in Equation (2).

(2) $$\theta (x,y,z)=\frac{{\left[{r(x,y,z)}^{2}-{R_{b}(x,y,z)}^{2}\right]}^{\frac{1}{2}}}{R_{b}(x,y,z)}-{tan}^{-1}\left[\frac{{\left[{r(x,y,z)}^{2}-{R_{b}(x,y,z)}^{2}\right]}^{\frac{1}{2}}}{R_{b}(x,y,z)}\right]$$

where θ(x,y,z) is the vectorial angle at a specific transformed coordinate, r(x,y,z) is the radius at a specific transformed coordinate with reference to the base circle, and $R_{b}(x,y,z)$ is the base circle radius at a specific transformed coordinate.

To fully separate the distinct waviness component from the raw data on rolled surface length, a Gaussian filter is applied [63,64,65,66]. This crucial process requires a proper specification of filtering parameters, primarily the cut-off wavelength, $\lambda_{c}$, based on the expected roughness topography. For a specified cut-off wavelength, the Gaussian weight function, s, at any surface point, can be described as follows:

(3) $$s(x,y)=\frac{1}{\alpha^{2}\cdot \lambda_{c}^{2}}exp\left\{-\pi \left[\frac{x^{2}+y^{2}}{{\left(\alpha \cdot \lambda_{c}\right)}^{2}}\right]\right\}$$

where x and y are the coordinates of a surface point, and α is a constant value amounting to approximately 0.4697, designated to ensure 50% transmission at the cut-off. The filtered waviness, or, particularly, mean surface, z(x,y), is obtained by:

(4) z(x,y)=p(x,y)·s(x,y)

where p(x,y) is the raw surface. The mean surface can be further described as:

(5) z(x,y)=∬p(ξ,η)·s(x−ξ,y−η)dξdη

where p(ξ,η) is the filtered weight at the offset ξ,η. The narrated procedure, describing the direct convolution of the primary surface p(x,y) with a Gaussian kernel s(x,y), extracts the surface roughness, r(x,y), as expressed by:

(6) r(x,y)=p(x,y)−z(x,y)

The cut-off wavelengths, $\lambda_{c}$, used in the study, are specified according to the expected flank surface roughness for each manufacturing method and gear module. Accordingly, a typical value of 0.8 mm is assigned to hobbed POM-C gears, which fit most standard-precision industrial plastic gears, while a value of 2.5 mm is assigned to MEX PPS-CF and SLS PA 12 gears, corresponding to coarse 3D-printed surfaces.

**Figure 3:**
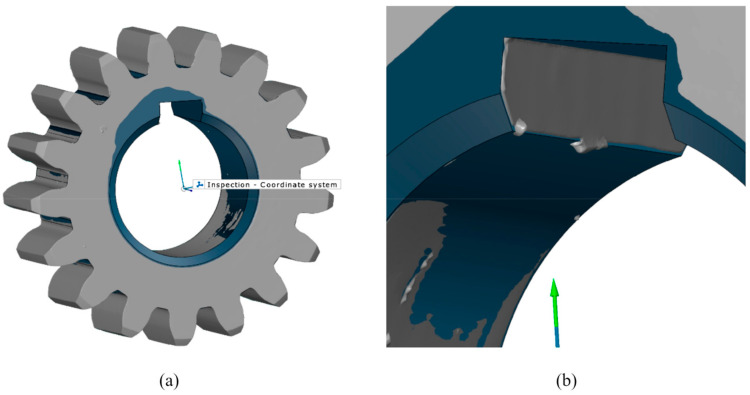
Overlap entity: (**a**) pre-alignment of the point cloud and CAD model by standard fitting; (**b**) best-fit alignment at the reference keyway grooves.

Data processing and alignment were performed using Zeiss Inspect 2023 (Carl Zeiss GOM Metrology GmbH, Braunschweig, Germany), using a local best-fit algorithm to evaluate profile and lead deviations relative to the nominal CAD geometry [67,68]. Within the scope of this study, lead deviations, as the most influential characteristics of tolerance quality, are evaluated by comparing the scanned data model to a reference dataset of an ideal computer-aided design (CAD) model, as presented in Figure 3a.

The proper assessment of geometric deviation requires pre-alignment of both models using a standard fitting method to achieve a high-quality overlap [69,70]. As the final entity needs to be viable for fine-precision measurements, an additional local best-fit alignment is applied to the holes and keyway grooves of both the scanned areal surface data and the CAD model, which are the most vital geometric features in gear mounting, as shown in Figure 3b.

**Figure 4:**
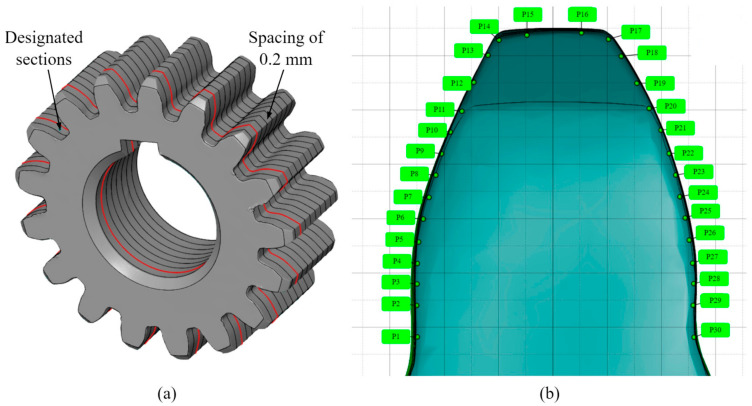
Post-processing of the scanned data: (**a**) designated planar sections spaced 0.2 mm apart; (**b**) single-tooth deviations at 30 points ranging from the root to the tip diameter.

To precisely determine the tolerance quality of gear specimens manufactured via different methods, areal data are obtained from multiple flank-surface points across a set of equidistant planar sections, thereby providing insight into geometric deviations in the lead directions, which are vital for a comprehensive understanding of the distinct qualities of the manufacturing methods used. The methodology employed in this study comprises 11 planar sections spaced at 0.2 mm intervals, as illustrated in Figure 4a. Geometric deviations for each tooth are determined at 30 specific points extending from the root to the tip diameter, including 28 points on the flanks and 2 points on the tooth tip (Figure 4b).

## 3. Results and Discussion

### 3.1. Surface Roughness

**Figure 5:**
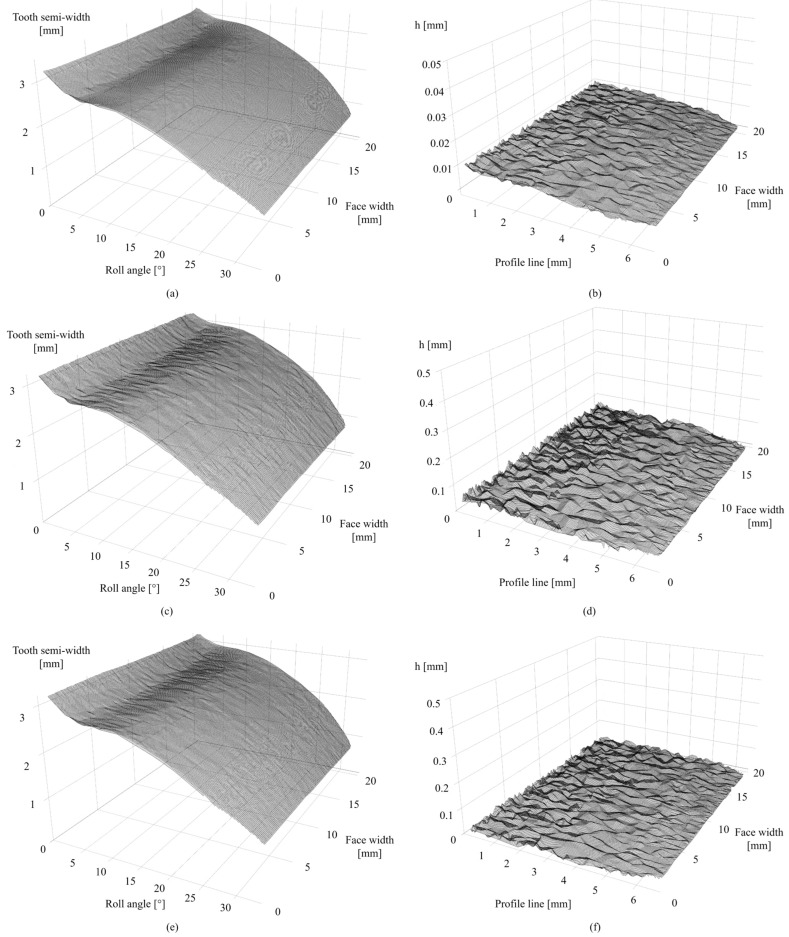
Surface roughness evaluation: (**a**) flank surface of hobbed POM-C gear; (**b**) surface roughness of hobbed POM-C gear; (**c**) flank surface of MEX PPS-CF gear; (**d**) surface roughness of MEX PPS-CF gear; (**e**) flank surface of SLS PA 12 gear; (**f**) surface roughness of SLS PA 12 gear.

Figure 5a illustrates the single-tooth flank surface of the hobbed POM-C gear, with its corresponding surface roughness topography visualized in Figure 5b (*S*_a_ = 5 µm). The representative flank of the MEX PPS-CF gear, depicted in Figure 5c, displays a distinct surface contrast relative to the hobbed specimen. The areal roughness map in Figure 5d confirms a significantly higher arithmetic mean height of 25 µm for the MEX process. This comparison highlights that the surface topographies produced by hobbing and MEX 3D printing differ by an order of magnitude, reflecting the divergent precision of these manufacturing routes. Furthermore, Figure 5e displays the tooth flank of the PA 12 gear produced via SLS. Although this surface exhibits improved characteristics over the MEX method due to the uniform grain structure of the sintered powder, it fails to match the refined smoothness of the hobbed surface, yielding an *S*_a_ value of 12 µm (Figure 5f). The comprehensive dataset, including all areal surface parameters defined per ISO 25178, is summarized in Table 4.

The surface roughness values reported in Table 4 were computed using the ‘Surfalize’ Python module (ver. 0.16.7, Fraunhofer IWS, Dresden, Germany) for areal and periodic parameter analysis. The module directly implements the ISO 25178 standard for surface texture characterization, utilizing the transformed surface area data as its primary input.

The comparative analysis of the surface topography parameters in Table 4 reveals distinct topographical signatures associated with each manufacturing technology. The hobbed POM-C gear exhibited the most refined surface finish, with arithmetic mean height (*S*_a_) and maximum height (*S*_z_) both measured at 5.0 µm. This high level of precision is further supported by a high homogeneity index (>0.7) and a remarkably short period of 7.0 µm, corresponding to the fine tool marks left by the hobbing cutter. In contrast, the MEX-printed PPS-CF gear exhibited the highest surface irregularity, with a *S*_a_ of 25 µm and a maximum height (*S*_z_) of 119.1 µm.

This significant vertical deviation, coupled with a dominant period of 144.3 µm, is a direct consequence of the “stair-stepping” effect and the deposition of discrete filament beads characteristic of the MEX process. The SLS-printed PA 12 gear occupied a middle ground, featuring an *S*_a_ of 12 µm and an *S*_z_ of 33.3 µm. While powder-based fusion in SLS offers a more isotropic, smoother texture than MEX, the grainy nature of sintered particles prevents it from achieving the smoothness of traditional machining. Interestingly, the study associates a higher interfacial area ratio (*S*_dr_) for POM-C (73.3%) compared to PPS-CF (11.2%) and PA 12 (29.7%). The study suggests that gears manufactured via MEX, concerning the specific PPS-CF gear under the presented context, may encounter challenges in maintaining uniform lubrication films compared to their hobbed or SLS counterparts, based on the extreme maximum valley depth (*S*_v_) of 73.9 µm.

### 3.2. Tolerance Quality

Comprehensive datasets containing detailed measurements for all specimens are provided in the Appendix A (Table A1, Table A2 and Table A3).

**Figure 6:**
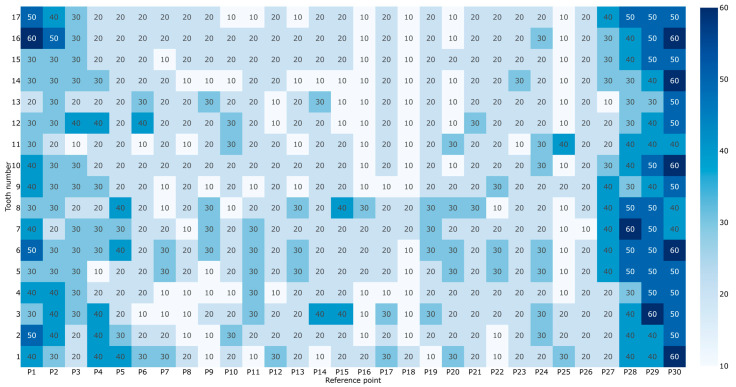
Measured geometric deviations of the hobbed POM-C gear specimens.

The POM-C gear demonstrates the highest level of precision among the tested specimens, consistently achieving the quality grades Q9 and Q10. The mean deviations across all 17 measured teeth are remarkably stable, ranging from 20.6 µm to 28.6 µm. Even at the “worst” reference points (typically P1 near the tip or P30 near the root), the maximum recorded deviation rarely exceeds 60 µm (Table A1). This indicates that the mechanical hobbing process maintains excellent involute profile control, which is essential for reducing transmission errors and noise. The total mean deviation is 23.62 µm, and the standard deviation of 2.18 µm indicates that the data points are relatively close to the mean, with approximately 68% of the measured values falling between 21.44 µm and 25.8 µm. A detailed graphical preview of the deviations of the hobbed POM-C gear, at each tooth for 30 reference points, is presented in Figure 6.

**Figure 7:**
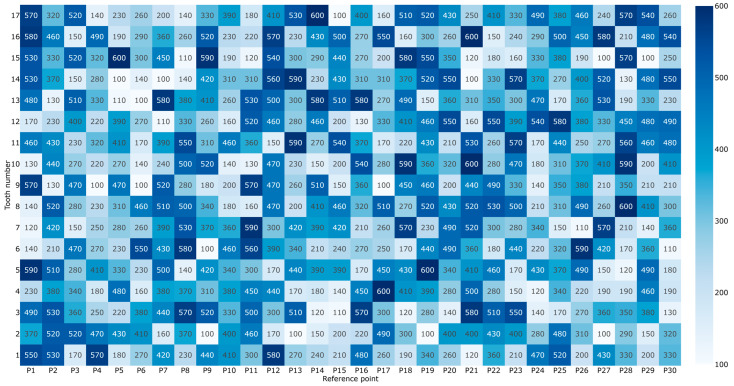
Measured geometric deviations of the MEX-printed PPS-CF gear specimens.

The MEX-printed PPS-CF gear exhibited the poorest performance, with a quality grade lower than Q12. Mean deviations are significantly higher, ranging from 312 µm to 378.3 µm. In some specific points (e.g., P1 on Tooth 5), deviations spike as high as 590 µm (Table A2). While the PPS-CF material offers high thermal and mechanical strength, the presence of carbon fibers can introduce localized cooling variations and nozzle drag, leading to substantial geometric inaccuracies, as shown in Table 5. The mean deviation is 344.88 µm, with a standard deviation of 19.6 µm. For the specific data, about 68% of the values fall within the range of 325.28 µm to 364.48 µm. These gears would likely require post-machining to be viable for high-speed drivetrain applications. Detailed imaging of MEX-printed PPS-CF gear deviations is displayed in Figure 7.

**Figure 8:**
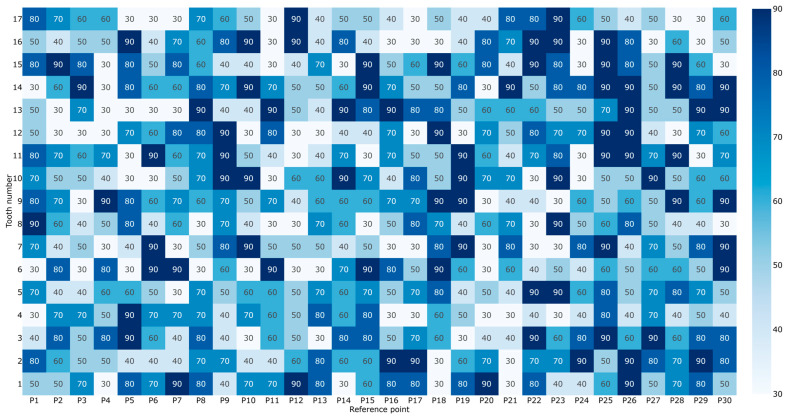
Measured geometric deviations of the SLS PA 12-printed PA 12 gear specimens.

The SLS PA 12 powder gear represents a significant step down in precision from hobbing but remains functionally superior to MEX. With a quality grade of Q11, these gears are suitable for many industrial applications. The average deviations range from 52.3 µm to 68.0 µm, roughly double those of the hobbed specimen (Table A3). Unlike MEX, the SLS process shows relatively uniform deviations across the tooth profile. The lack of support structures in the SLS powder bed likely contributes to better geometric preservation compared to extrusion-based methods. The total mean deviation in this case amounts to 59.32 µm, with a somewhat higher standard deviation of 4.29 µm, and 68% of the values range from 325.88 µm to 364.48 µm. A detailed preview of the deviations of the SLS PA 12 powder gear is displayed in Figure 8.

The data in Table 5 summarizes the measured quality grades and highlights a significant “accuracy gap” between subtractive and additive manufacturing for polymers.

The preliminary experimental results reveal a clear hierarchy in manufacturing quality, with conventional hobbing of POM-C significantly outperforming additive manufacturing (AM) methods. The hobbed POM-C gear achieved the highest precision, reaching accuracy grades of Q9 to Q10 and the lowest surface roughness at 5 μm. In contrast, the SLS-printed PA 12 gear ranked second with a Q11 grade and an intermediate roughness of 12 μm, while MEX-printed PPS-CF exhibited the lowest precision (falling below the Q12 grade) and the highest roughness at 25 μm.

### 3.3. Limitations of the Current Study and Future Research

Even though a high-precision GOM Scan 1 3D scanner can be used for the characterization of surface topography, the applicability of point cloud data for the assessment of surface roughness is conditioned by the roughness scale and the scanner’s spatial resolution. The main biases in the presented measuring methodology arise from the scanner’s lateral resolution and optical spot size, averaging the features of small surfaces and smoothing out peak heights and valley depths that fall below the scanner’s resolution, leading to the systematic attenuation of micro-scale roughness parameters.

Although a proposed point cloud-based 3D scanning methodology is applicable for evaluating surface roughness parameters and tolerance grades, a thorough study involving multi-component measurements is necessary, as any conclusion must be generalized in light of the data dispersion arising from manufacturing process variability. Likewise, further research should address common polymer materials used with the versatile manufacturing methods, as the disparities in surface roughness and tolerance grades observed in the study are affected by the different thermo-mechanical properties of the materials, resulting in differences in processing behavior, cooling, shrinkage characteristics, and distortion-related settings.

Additionally, as the present work does not constitute a full metrological validation against any traceable reference measurement technique, future work should validate this methodology through a comparative study against high-precision tactile (stylus) profilometry. Regarding filtering, specifically the cut-off wavelengths ($\lambda_{c})$, a sensitivity analysis is suggested for new materials. This ensures that Gaussian filtering does not smooth critical surface asperities, which would lead to an underestimation of roughness. Sampling density must be optimized relative to the cut-off length to avoid aliasing and artifacts that could result in unusable data.

The tolerance grades established experimentally for MEX and SLS components in this study could be applied in any future design of additively manufactured assembly fixtures used in automotive production. As an illustrative example, a locating pin or conforming nest fabricated via MEX to position a powertrain bracket during assembly may exhibit a mean profile deviation of 0.3–0.6 mm—consistent with the MEX PPS-CF results recorded under the specific study conditions—which could likely translate directly into a positional uncertainty of the same order at the contact interface—a deviation that may exceed the assembly tolerance budget for precision subassemblies such as engine mounts or transmission housings [11,71]. In a comparable future scenario, SLS PA 12 components with achieved Q11 tolerance grade and mean deviations of 0.08–0.15 mm, under the presented settings, could be employed for secondary locating surfaces with less stringent positional repeatability requirements. This experimental differentiation can further yield a data-driven basis for technology selection in adaptive tooling design, whereby SLS would be preferable for surfaces with direct dimensional function, while MEX would be reserved for suitable non-critical clamping bodies, covers, or ergonomic handles [12,13].

From a surface topography perspective, the *S*_a_ values measured for MEX components (*S*_a_ > 10 µm) indicate a contact surface condition that could lead to inconsistent friction behavior under the clamping loads typical of assembly fixtures, potentially causing micro-slippage of the located part during fastening operations [14,22]. The hobbed POM-C data (*S*_a_ < 5 µm), for the specific material and tested conditions, establishes a reference baseline for precision-grade contact surfaces, against which the surface quality of additively manufactured alternatives may be evaluated. For any future adaptive tooling applications, the present findings suggest that MEX contact surfaces may benefit from post-processing—such as chemical smoothing or machining of functional faces—to bring Sa into the 1–3 µm range—a condition consistent with reliable frictional contact in assembly jigs. The 3D scanning methodology presented within this study could efficiently detect these distinct surface states across the full flank geometry, making it applicable as an inline quality-control procedure for incoming tooling inspection prior to line deployment.

## 4. Conclusions

This study present a foundation for the comparative analysis of polymer gear manufacturing methods, examining the distinct characteristics and quality outcomes associated with the conventional hobbing for polyoxymethylene copolymer (POM-C) bar stock and two AM methods employed: Material Extrusion (MEX) using a 100% infill for carbon fiber reinforced polyphenylene sulfide (PPS-CF), and Selective Laser Sintering (SLS) for polyamide 12 (PA 12).

Surface roughness values were derived using the Python 3.13 ‘Surfalize’ module, which implements the ISO 25178 standard for areal surface texture analysis. The roughness, within the materials and associated process conditions presented in the study, varied significantly across methods: the hobbed POM-C gear exhibited the smoothest surface (*S*_a_ = 5 µm), whereas the MEX PPS-CF gear showed a significantly rougher profile (*S*_a_ = 25 µm). The SLS PA 12 gear, characterized by the grainy texture of sintered powder, achieved a mid-range roughness of (*S*_a_ = 12 µm), failing to match the smoothness of the hobbed surfaces for the specific material-processing conditions.

Geometric deviations and accuracy grades were evaluated in accordance with DIN 3961. Under the examined settings, the hobbed POM-C gears achieved a quality range of Q9 to Q10. In contrast, the MEX-printed PPS-CF gears fell outside the Q12 quality grade due to substantial geometric deviations. These results suggest three primary conclusions, non-generalizable to other materials and process settings:

- For the configurations and material parameters examined in this study, polymer gear produced by conventional hobbing had smoother surfaces and a higher tolerance quality compared to the tested MEX and SLS configurations.
- Integrating high-resolution optical scanning with advanced data processing provides a framework for evaluating geometrical deviations in polymer gears. Using a GOM Scan 1 3D scanner with blue structured light, the methodology captured dense point clouds (exceeding 6 million points), ensuring fine surface details and roughness were resolved.
- The implementation of a two-stage alignment process—combining initial fitting with local best-fit on critical features—minimized positioning errors when comparing scanned meshes to ideal CAD models. Ultimately, the use of sectional analysis across 11 planar sections enabled the precise determination of lead and profile deviations, demonstrating that non-contact optical methods have a high potential for the quality assessment of complex polymer gear geometries.
- Future research should repeat the conducted experiment with a larger number of gears to confirm the repeatability of the results.

## Figures and Tables

**Table 1:** Comparison of material properties and production settings for POM-C, PPS-CF, and PA 12 gear specimens.

Feature	POM-C (Polyoxymethylene)	PPS-CF (Carbon Fiber Reinforced Polyphenylene Sulfide)	PA 12 (Polyamide 12)
Technology	Subtractive (hobbing)	Additive, MEX (material extrusion)	Additive, SLS (selective laser sintering)
Method	Conventional machining with a hob cutter	3D printing (extrusion) with 100% infill	3D printing (powder sintering)
Raw Material	Extruded stock rods	Filament	Powder

**Table 2:** Summary of material specifications, geometric parameters, and associated test standards for gear specimens.

Material/Geometric Properties	POM-C		PPS-CF		PA 12	
	Value	Method	Value	Method	Value	Method
Tensile modulus	2800 MPa	EN ISO 527-2 [40]	4376 ± 72 MPa ^1^ 7766 ± 166 MPa ^2^ 2392 ± 114 MPa ^3^	ASTM D3039/D3039M-08 [41]	1850 MPa	ASTM D638-14 (sample T1) [42]
Tensile stress at yield	67 MPa	EN ISO 527-2	47.5 ± 1.9 MPa ^1^ – –	ASTM D3039/D3039M-08	50 MPa	ASTM D638-14 (sample T1)
Elongation at break	32%	EN ISO 527-2	2.0 ± 0.1%^1^ 2.2 ± 0.1%^2^ 1.1 ± 0.2%^3^	ASTM D3039/D3039M-08	11% 11% 6%	ASTM D638-14 (sample T1)
Flexural strength	91 MPa	EN ISO 178 [43]	87.0 ± 1.2 MPa ^1^ 95.2 ± 0.6 MPa ^2^ 56.3 ± 0.8 Mpa ^3^	EN ISO 178	66 MPa	ASTM D790-15 [44]
Flexural modulus	2600 MPa	EN ISO 178	5106 ± 75 MPa ^1^ 6175 ± 96 MPa ^2^ 1886 ± 51 MPa ^3^	EN ISO 178	1600 MPa	ASTM D790-15
Notched izod	60 J/m	ISO 180 [45]	4.8 ± 0.2 kJ/m^2^	ISO 179-1 [46]	32 J/m	ASTM D256-10 [47]
Heat deflection temp.	100 °C	ISO 75-2 [48]	104 °C	ISO 75-2	87 °C	ASTM D648-18 [49]
Vicat softening temp.	150 °C	ISO 306 [50]	>230 °C	ISO 306	175 °C	ASTM D1525-17e1 [51]
Water absorption	0.05%	ISO 22007-4 [52]	0.05%	ISO 62 [53]	0.66%	ASTM D570-98(2010)e1 [54]
Profile type	Involute ISO 53 profile A					
Teeth number	17					
Face width	20 mm					
Modulus	3 mm					
Normal pressure angle	20°					
Tip diameter	57 mm					
Reference diameter	51 mm					
Root diameter	44.4 mm					
Base circle diameter	47.9 mm					
Profile shift coefficient	0					
Profile line length	6.67 mm					

^1^ xy (flat) plane; ^2^ yz (side) plane; ^3^ z (upright) direction.

**Table 3:** Key technical and metrological data for the GOM Scan 1 3D scanner.

Specification/Metrological Parameter	Value
Points per scan	1 × 10^6^
Point distance	0.06 mm
Measuring area	200 × 125 mm^2^
Working distance	450 mm
Repeatability	0.02–0.03 mm
Dimensional uncertainty	0.03–0.05 mm
Mesh resolution influence	0.1–0.2 mm
Alignment error	0.01–0.05 mm ^1^
	0.02–0.08 mm ^2^
	0.02–0.1 mm ^3^
Preprocessing sensitivity	5–30 µm ^4^
	5–20 µm ^5^
	10–30 µm ^6^
	10–50 µm ^7^

^1^ best-fit alignment; ^2^ reference point system (RPS) 3-2-1; ^3^ feature alignment (holes/planes); ^4^ scanning spray thickness; ^5^ marker center detection error; ^6^ calibration drift; ^7^ surface reflectivity noise.

**Table 4:** Areal surface roughness parameters for the hobbed, MEX, and SLS gear specimens.

Surface Roughness Parameters	POM-C	PPS-CF	PA 12 Powder
Arithmetic mean height, *S*_a_	5.0 µm	25.0 µm	12.0 µm
Root mean square height, *S*_q_	6.1 µm	29.1 µm	13.4 µm
Maximum peak height, *S*_p_	3.5 µm	45.2 µm	11.2 µm
Maximum valley depth, *S*_v_	1.5 µm	73.9 µm	22.1 µm
Maximum height, *S*_z_	5.0 µm	119.1 µm	33.3 µm
Interfacial area ratio, *S*_dr_	73.3%	11.2%	29.7%
Period, *Λ*	7.0 µm	144.3 µm	42.3 µm
Homogeneity, *H*	>0.7	>0.5	>0.6

**Table 5:** Measured geometric deviations and corresponding tolerance quality grades for POM-C, PPS-CF, and PA 12 gears.

Manufacturing Method	Quality Grade (DIN 3961)	Avg. Deviation Range	Mean Deviation Value	Standard Deviation (SD)	Primary Constraint
Hobbing (POM-C)	Q9–Q10	20–29 µm	23.62 µm	2.18 µm	Thermal stability of stock
MEX (PPS-CF)	< Q12	312–378 µm	344.88 µm	19.6 µm	Layer height/nozzle diameter
SLS (PA 12)	Q11	52–68 µm	59.32 µm	4.29 µm	Powder particle size/sintering

## Data Availability

The original contributions presented in this study are included in the article. Further inquiries can be directed to the corresponding authors.

## Appendix A

### Appendix A.1

Table A1 presents the geometrical deviations and DIN 3961 tolerance grades for the POM-C gears.

**Table A1:** Geometrical deviations and tolerance quality of hobbed POM-C gears.

Point	Geometrical Deviations per Tooth (µm)																
	1	2	3	4	5	6	7	8	9	10	11	12	13	14	15	16	17
P1	40	50	30	40	30	50	40	30	40	40	30	30	20	30	30	60	50
P2	30	40	40	40	30	30	20	30	30	30	20	30	30	30	30	50	40
P3	20	20	30	30	30	30	30	20	30	30	10	40	20	30	30	30	30
P4	40	40	40	20	10	30	30	20	30	20	20	40	20	30	20	20	20
P5	40	30	20	20	20	40	30	40	20	20	20	20	20	20	20	20	20
P6	30	20	10	20	20	20	20	20	20	20	10	40	30	20	20	20	20
P7	30	20	10	10	30	30	20	10	10	20	20	20	20	20	10	20	20
P8	20	10	10	10	20	20	10	20	20	20	10	20	20	10	20	20	20
P9	10	10	20	10	10	30	30	30	10	20	20	20	30	10	20	20	20
P10	20	30	20	10	20	20	20	10	20	20	30	30	20	10	20	20	10
P11	10	20	30	30	30	30	30	20	10	20	20	20	20	20	20	20	10
P12	30	20	20	10	20	20	20	20	20	20	20	10	10	20	20	20	20
P13	20	20	20	20	30	30	20	30	10	20	10	20	20	10	20	20	10
P14	10	20	40	20	20	20	20	20	20	20	20	20	30	10	20	20	20
P15	20	20	40	20	20	20	20	40	20	20	20	10	10	10	20	20	20
P16	20	10	10	10	20	20	20	30	10	10	10	10	10	10	10	10	10
P17	30	20	30	10	20	20	20	20	10	20	20	20	20	20	20	20	20
P18	20	10	10	10	10	10	20	20	10	10	10	10	10	10	10	10	10
P19	10	20	30	20	20	30	30	30	20	20	20	20	20	20	20	20	20
P20	30	20	20	20	30	30	20	30	20	10	30	10	10	10	20	10	10
P21	20	20	20	20	20	20	20	30	20	20	20	30	20	20	20	20	20
P22	10	10	20	20	30	30	20	10	30	20	20	20	20	20	20	20	20
P23	20	20	20	20	20	20	20	20	20	20	10	20	20	30	20	20	20
P24	20	30	30	20	30	30	20	20	20	30	30	20	20	20	20	30	20
P25	30	20	20	10	10	10	10	10	20	10	40	10	10	10	10	10	10
P26	20	20	20	20	20	20	10	20	20	20	20	20	20	20	20	20	20
P27	20	20	20	20	40	40	40	40	40	30	20	20	10	30	30	30	40
P28	40	40	40	30	50	50	60	50	30	40	40	30	30	30	40	40	50
P29	40	40	60	50	50	50	50	50	40	50	40	40	30	40	50	50	50
P30	60	50	50	50	50	60	40	40	50	60	40	50	50	60	50	60	50
Mean	23.3	24	26	21.3	25.3	28.6	25.6	25.6	22.3	23.6	21.7	23	20.6	21	22.6	25.3	21.7
Standard deviation (SD): 2.18 µm																	
Quality grade (DIN 3961): Q9 to Q10																	

### Appendix A.2

The data on geometrical deviations and corresponding tolerance quality for MEX PPS-CF gears are presented in Table A2.

**Table A2:** Geometrical deviations and tolerance quality of MEX PPS-CF gears.

Point	Geometrical Deviations per Tooth (µm)																
	1	2	3	4	5	6	7	8	9	10	11	12	13	14	15	16	17
P1	550	370	490	230	590	140	120	140	570	130	460	170	480	530	530	580	570
P2	530	520	530	380	510	210	420	520	130	440	430	230	130	370	330	460	320
P3	170	520	360	340	280	470	150	280	470	270	230	400	510	150	520	150	520
P4	570	470	250	180	410	270	250	230	100	220	320	220	330	280	320	490	140
P5	180	430	220	480	330	230	280	310	470	270	410	390	110	100	600	190	230
P6	270	410	380	160	230	550	260	460	100	140	170	270	100	140	300	290	260
P7	420	160	440	380	500	430	390	510	520	240	390	110	580	100	450	360	200
P8	230	370	570	370	140	580	530	500	280	500	550	330	380	140	110	260	140
P9	440	100	520	310	420	100	370	340	180	520	310	260	410	420	590	520	330
P10	410	400	330	380	340	460	360	180	200	140	460	160	260	310	190	230	390
P11	300	460	500	450	300	560	590	160	570	130	360	520	530	310	120	220	180
P12	580	170	300	440	170	390	300	470	470	470	150	460	500	560	540	570	410
P13	270	100	510	170	440	340	420	200	260	230	590	280	300	590	300	230	530
P14	240	150	120	180	390	210	390	410	510	150	270	460	580	230	290	430	600
P15	210	200	110	140	390	240	420	460	150	200	540	200	510	430	440	500	100
P16	480	220	570	450	170	270	210	320	360	540	370	130	580	310	270	270	400
P17	260	490	300	600	450	250	260	510	100	280	170	330	270	310	200	550	160
P18	190	300	120	410	430	170	570	270	450	590	220	410	490	370	580	160	510
P19	340	100	280	390	600	440	230	520	460	360	430	460	150	520	550	300	520
P20	260	400	140	280	340	490	490	430	200	320	210	550	360	550	350	260	430
P21	120	400	580	500	410	360	520	520	440	600	530	160	310	100	120	600	250
P22	360	430	510	280	460	180	300	530	490	280	260	550	350	330	180	150	410
P23	210	400	550	150	170	440	280	500	330	470	570	390	300	570	160	240	330
P24	470	280	140	120	430	220	340	210	140	180	170	540	470	370	330	290	490
P25	520	480	170	340	370	320	150	310	350	310	440	580	170	270	380	500	380
P26	200	310	270	220	490	590	110	490	380	370	250	380	360	400	190	450	460
P27	430	100	360	190	150	420	570	260	210	410	270	330	530	520	100	580	240
P28	330	290	350	190	120	170	210	600	350	590	560	450	190	130	570	210	570
P29	200	150	380	460	490	360	140	410	210	200	460	480	330	480	100	480	540
P30	330	320	130	190	180	110	360	300	210	410	480	490	230	550	250	540	260
Mean	335.7	316.7	349.3	312	356.7	332.3	333	378.3	322	332	367.7	356.3	360	348	332	368.7	362.3
Standard deviation (SD): 19.6 µm																	
Quality grade (DIN 3961): less accurate than Q12																	

### Appendix A.3

Finally, data on geometrical deviations and tolerance quality of the SLS PA 12 powder gears are presented in Table A3.

**Table A3:** Geometrical deviations and tolerance quality of SLS PA 12 powder gear.

Point	Geometrical Deviations per Tooth (µm)																
	1	2	3	4	5	6	7	8	9	10	11	12	13	14	15	16	17
P1	50	80	40	30	70	30	70	90	80	70	80	50	50	30	80	50	80
P2	50	60	80	70	40	80	40	60	70	50	70	30	30	60	90	40	70
P3	70	50	50	70	40	30	50	40	30	50	60	30	70	90	80	50	60
P4	30	50	80	50	60	80	30	50	90	40	70	30	30	30	30	50	60
P5	80	40	90	90	60	30	40	80	80	30	30	70	30	80	80	90	30
P6	70	40	60	70	50	90	90	40	60	30	90	60	30	60	50	40	30
P7	90	40	40	70	30	90	30	60	70	50	60	80	30	60	80	70	30
P8	80	70	80	70	70	30	50	30	60	70	70	80	90	80	60	60	70
P9	40	70	40	40	50	60	80	70	70	90	90	90	40	70	40	80	60
P10	70	40	30	70	60	30	90	40	50	90	50	30	40	90	40	90	50
P11	70	40	60	60	60	90	50	30	70	30	40	80	90	70	30	30	30
P12	90	60	50	50	50	30	50	30	40	60	30	30	50	50	40	90	90
P13	80	80	30	80	70	30	50	70	60	60	40	30	40	50	70	40	40
P14	30	60	80	60	60	70	40	60	60	90	70	40	90	60	30	80	50
P15	60	60	80	80	70	90	50	30	60	70	30	40	80	90	90	40	50
P16	80	90	50	30	50	80	30	50	70	40	70	70	90	70	50	30	40
P17	80	90	70	30	70	50	30	80	70	80	50	30	80	50	60	30	30
P18	30	30	60	60	80	90	80	70	90	50	50	90	80	50	90	30	50
P19	80	60	30	50	40	60	90	40	90	90	90	30	50	80	60	40	40
P20	90	70	40	30	50	30	30	60	30	70	60	70	60	30	80	80	40
P21	30	30	40	30	40	60	80	70	40	70	40	50	60	90	40	70	80
P22	80	70	90	40	90	40	30	30	40	30	70	80	60	50	90	90	80
P23	40	70	60	30	90	50	30	90	30	90	80	70	50	80	80	90	90
P24	40	90	80	40	60	40	80	50	60	30	30	70	50	80	30	30	60
P25	60	50	90	80	80	60	90	60	50	50	90	90	70	90	90	90	50
P26	90	90	60	40	50	50	40	80	60	50	90	90	90	90	80	80	40
P27	50	80	90	70	70	60	70	50	50	90	70	40	50	50	50	30	50
P28	70	70	60	40	80	60	50	40	90	50	90	30	50	90	90	60	30
P29	80	90	80	50	70	50	80	40	60	60	30	70	90	80	60	30	30
P30	50	80	80	40	50	90	90	30	90	60	70	60	90	90	30	50	60
Mean	63.7	63.3	62.3	54	60.3	57.7	57	54	62.3	59.7	62	57	60.3	68	62.3	57.7	52.3
Standard deviation (SD): 4.29 µm																	
Quality grade (DIN 3961): Q11																	
